# Pathogenic bacterial profile, associated factors, and antimicrobial susceptibility patterns in intra-city public transport in Harar City, Eastern Ethiopia

**DOI:** 10.3389/fpubh.2025.1521479

**Published:** 2025-04-08

**Authors:** Nejib Sultan, Ayichew Seyoum, Firayad Ayele, Getachew Kabew Mekonnnen

**Affiliations:** School of Medical Laboratory Sciences, College of Health and Medical Sciences, Haramaya University, Harar, Ethiopia

**Keywords:** pathogenic bacterial load, antimicrobial susceptibility patterns, intra-city public transport, public transportation hygiene, Harar

## Abstract

**Background:**

Public transportation plays a vital role in urban settings, especially with the expansion of mass transit systems. However, concerns have arisen regarding the spread of antibiotic-resistant bacterial pathogens, primarily through contact with surfaces in public transport. There is limited data on this topic in Eastern Ethiopia, particularly in Harar. Therefore, this study aimed to assess the pathogenic bacterial profile, antimicrobial susceptibility patterns, and associated factors in intra-city public transport in Harar City, Eastern Ethiopia, from 20 June to 30 October 2023.

**Methods:**

A cross-sectional study was conducted involving 258 intra-city public transport vehicles selected through convenience sampling. The data collection process was conducted using structured questionnaires administered to drivers, and swab samples were obtained from frequently touched surfaces, such as seats, handles, and doors. These specimens were shipped to a laboratory within 2 h for microbiological analysis. Statistical analysis was performed using SPSS version 21. Linear and logistic regression analyses were employed to identify factors associated with pathogenic bacteria contamination, with statistical significance established at a *p*-value of <0.05 and a 95% confidence interval (CI).

**Results:**

A total of 258 vehicles were sampled, with pathogenic bacteria isolated from 192 vehicles, resulting in an isolation rate of 74.4% (95% CI: 69–80%). The mean colony-forming unit (CFU) count was 2.994 × 10^4^ (SD: ± 0.562 × 10^4^ CFU). The predominant pathogens identified included *coagulase-negative Staphylococci* (40.3%), *Staphylococcus aureus* (27.5%), *Klebsiella species* (11.6%), and *Escherichia coli* (10.9%). High antimicrobial resistance rates were observed, particularly against ampicillin (97.6%) and oxacillin (56.5%) in Gram-positive isolates, while Gram-negative rods exhibited varied resistance patterns. Multidrug resistance was detected in 22.5% of isolates. Significant factors associated with pathogenic bacteria contamination included sampling location (handles and seats), multiple routes, high passenger counts, unclean surfaces, and afternoon sampling.

**Conclusion:**

This study highlights alarming levels of bacterial contamination in public transport, the prevalence of pathogenic bacteria, and significant antibiotic resistance. Implementing effective hygiene measures and ensuring regular sanitation are essential to mitigating microbial risks and controlling the spread of community-acquired infections.

## Introduction

Transportation systems play a crucial role in facilitating the movement of passengers across different areas (1). Public transport services encompass a wide range of vehicles, from small animal-drawn transport to modern motorized vehicles (2–4). In Ethiopia’s capital city, Addis Ababa, 98% of the population relies on public transportation, with 26% being bus users (5). As a result, public transportation has become a highly shared space, frequently utilized by large numbers of people (6). Studies have reported higher levels of biological contaminants in public transportation settings (6, 7), with evidence suggesting that more than three-fourths of infections are transmitted through hand contact with contaminated surfaces or objects inside vehicles (8). Pathogenic bacteria commonly isolated from hand-touch surfaces include *Staphylococcus aureus, Streptococcus species, Escherichia coli, Vibrio cholera*, *Klebsiella species*, *Proteus species*, and *Salmonella species*. *Bacillus* sp., *Pseudomonas species*, and *Mycobacterium tuberculosis* (9). However, the bacterial profile may vary by location (10). Studies indicate that half of all travelers experience diarrhea caused by various bacteria (11). Additionally, approximately 80% of the human population is either permanently or transiently colonized with *S. aureus*, with an estimated 2% of the general population carrying *MRSA*, making everyone susceptible to these infections (12). The prevalence of *MRSA* isolates recovered from public transport settings has been reported to range from 1.5 to 36% (13).

According to the 2014 World Health Organization (WHO) survey report, there is a high rate of antibiotic resistance (79%) among bacteria causing common healthcare-associated infections (HCAIs) and community-acquired infections. Carbapenem-resistant and cephalosporin-resistant bacterial strains have emerged as major concerns, with multidrug-resistant (MDR) pathogens such as *Staphylococcus aureus, methicillin-resistant coagulase-negative Staphylococci (MRCoNS),* and *Enterobacteriaceae* posing significant threats (14). Similar to *MRSA*, antibiotic-resistant Gram-negative bacteria are increasingly recognized as severe public health threats. Notably, antibiotic-resistant *Enterobacteriaceae*, particularly colistin-resistant strains, extended-spectrum *β*-lactamase (ESBL)-producing strains, and carbapenem-resistant species, have rapidly spread worldwide, further exacerbating the global antibiotic resistance crisis (15).

The current high levels of contamination in public transportation systems put people at risk of emerging and drug-resistant strains of infectious diseases worldwide (16). The expansion of global transport networks has led to infectious disease pandemics and vector invasion events and highlighted the importance of vector-borne pathogens (17).

As a result, numerous diseases, such as tuberculosis, respiratory tract infections, diarrhea, and soft tissue infections, continue to affect large populations, particularly in Sub-Saharan Africa (18, 19). In line with this, more than 50% of travelers experience diarrhea caused by various bacteria (11). Furthermore, an estimated 80% of the human population is colonized by *S. aureus*, with 2% carrying *MRSA* (12). However, few studies focused on bacterial contamination in public transport, primarily targeting buses and specific pathogens such as MRSA have been undertaken in central Ethiopia.

Overall, there is limited data on public transport contamination, especially in Eastern Ethiopia. Therefore, this study aimed to determine the pathogenic bacterial load, profile, and antimicrobial susceptibility patterns in intra-city public transport and identify associated factors in Harar City, Eastern Ethiopia.

## Materials and methods

### Study design, period, and area

The cross-sectional study was conducted in Harar City, Ethiopia, from 20 June to 10 July 2023. Harar City is situated approximately 526 kilometers from Addis Ababa, with geographical coordinates of 90°20’ N and 42°0′10’ E (20). Harar was the capital city of the Harari region, which comprised six woredas and 19 kebeles, along with nine districts (woredas) encompassing 36 kebeles (21, 22). Since the majority (99.03%) of the population relies on intra-city public vehicles, Harar’s public transportation options include tricycles/Bajajs (*n* = 9,273), Ladas (*n* = 189), minibusses (*n* = 249), and busses (*n* = 27) (23). Moreover, Harar is a city in Ethiopia’s eastern region, where the country’s trade and business center is located; thus, it is densely populated with people from virtually all ethnicities, professions, and social classes.

### Study participants

The source population for this study included all intra-city public transport vehicles and their drivers operating in Harar City. The study population specifically focused on the intra-city public transport vehicles that provided services in Harar City during the data collection period.

### Inclusion and exclusion criteria

All intra-city public transport vehicles working in Harar City during the study period were included. Vehicles that were undergoing maintenance and not present in the waiting area during this time were excluded.

### Sample size determination and sampling technique

**Figure 1:**
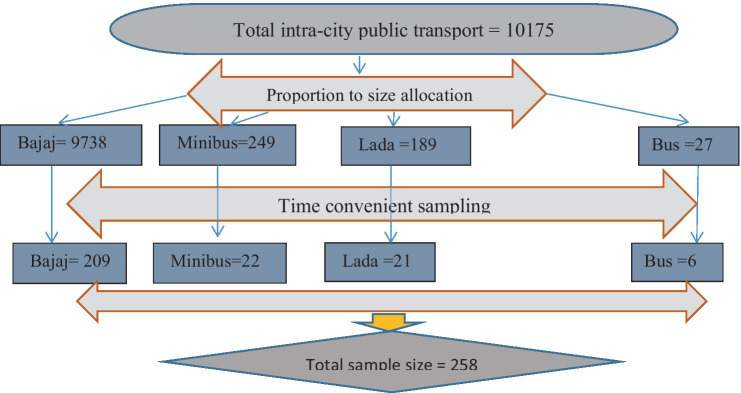
Time-convenient sampling on frequently touched surfaces of intra-city public transport at Harar, Eastern Ethiopia from June 20 to October 30, 2023.

The sample size for assessing contamination by pathogenic bacteria in intra-town public transport was determined using a single-population proportion formula (*n* = *Z*^2^ (1-*P*) *P*/*d*^2^). With a 95% confidence level and a 5% margin of error, and considering a prevalence rate of pathogenic bacterial contamination on bus surfaces in Mekelle Town (22%) (1), the calculated sample size was 258. The four public transport stations or bus stops were randomly selected from the nine available in Harar City, specifically Aretagna/Iman Masjid, Shell/Ageeb area, Station/Shewa Bar, and Jegol/Feres Magala. The sample size was then distributed proportionally based on the number of seats for each type of public transport in the town, allocating 209 samples for tricycles/Bajaj, 22 for minibusses, 21 for Lada vehicles, and 6 for busses. The swab samples were conveniently collected from various parts of the selected public transport vehicles (Figure 1).

### Data collection methods and procedure

#### Data collection and sample collection techniques

Data were collected by two qualified laboratory professionals using a structured questionnaire adapted from a similar study, which was later pretested. The questionnaire was prepared in English and translated into local languages (Afan Oromo and Amharic) to gather information on key variables such as sociodemographic traits, taxi types, sample locations, surface types, passenger counts, and service routes (multi-routes, hospital routes, sample times, and vehicle hygiene practices).

After obtaining voluntary consent from participants, triplet samples were collected from frequently touched areas of public transport: seats, handles, armrests, and door surfaces of each vehicle. According to existing environmental microbiology laboratory manuals (24), a marked area of 2 cm x 4 cm was swabbed with a sterile, moistened cotton swab, and the swabs were then placed in a sterile container with sterile buffered peptone water (1 mL). Additionally, a moistened swab was incubated alongside each batch of sampled swabs for quality control. The collected samples were transported promptly under cold chain conditions to the microbiological laboratory at Haramaya University College of Health and Medical Sciences within 2 h of collection.

### Bacterial colony count, isolation and identification

Surface swab specimens were inoculated onto MacConkey agar, blood agar, and 1 in 10 fourfold diluted nutrient agar. The inoculated agar plates were then incubated at 35°C ± 2 for 24 to 48 h. After the incubation period, the growth on the agar plates was examined to isolate and identify the bacteria present (25), and the viable bacterial load in the vehicles was estimated as colony-forming units per square centimeter of surface (CFU/cm^2^). Bacterial identification is based on colony characteristics, hemolytic appearance, differential properties of the culture media, and Gram reaction. Identification was performed using various standard biochemical tests, including catalase, oxidase, urease, indole, citrate utilization, glucose and lactose fermentation, gas and H2S production, lysine decarboxylation, and motility tests (5, 9, 26).

### Antibiotic susceptibility test

The antibiotic susceptibility patterns of the identified pathogenic bacterial species were determined using the standard Modified Kirby-Bauer disk agar diffusion method on Mueller Hinton agar, following the guidelines provided by the Clinical and Laboratory Standards Institute (CLSI 2022). A McFarland standard equivalent inoculum of bacteria was swabbed onto the surface of a Mueller Hinton agar plate for antibiotic susceptibility testing. Filter paper disks containing specific antimicrobial agents—ampicillin (10 μg), oxacillin (30 μg), ciprofloxacin (5 μg), gentamicin (10 μg), chloramphenicol (2 μg), ceftriaxone (30 μg), cefuroxime (30 μg), and erythromycin (15 μg)—were placed on the agar surface. Following overnight incubation, the diameter of the zone of inhibition around each disk was measured using the standardized tables provided by CLSI 2022, and a semi-qualitative report of susceptibility or resistance to each antibiotic was determined (27, 28).

### Operational definitions

The antimicrobial susceptibility pattern reflects how isolated bacteria respond to commonly prescribed drugs, indicating whether they are susceptible or resistant. This pattern is measured by comparing the zone of inhibition against a standard comparison chart, allowing for the interpretation of susceptible, intermediate, and resistant categories (5).

Hygienic practices: these practices refer to the cleaning protocols implemented at each car wash, evaluated with a yes/no response for the following: 1. dry wiping, 2. water only, 3. water and detergents, and 4. water and bleach.

Hospital route: were the vehicles designated as running hospital or non-hospital on the day of sampling (29)?

The sample: It includes the vehicle’s seats, handles (rail), and doors.

The level of pathogenic bacterial isolates: this is determined by counting the colonies and comparing them to WHO standards.

Multiple routes: vehicles were categorized as servicing multiple routes if they served two or more routes on the sampling day, while single-route vehicles served only one route (29).

Multidrug resistance: this is defined as the resistance of an isolate to three or more classes of antibiotics.

A pathogenic bacterial profile: it includes any health-important bacteria. If these bacteria grow on selective media, they are measured as yes/no and confirmed by biochemical tests.

Types of vehicles: there are various types of vehicles, such as: (1) minibusses, (2) Lada/Peugeot, (3) tricycles/Bajaj, and (4) busses; everyone has the right to use them, provided they adhere to the rules governing their use.

Types of surfaces: they include vehicle surfaces such as metals, plastics (such as polyvinyl), and cloth coverings for various vehicle parts.

### Data quality assurance

The questionnaire used in the study underwent a translation process to ensure consistency with the local languages Amharic and Afan Oromo. Initially, professional linguists translated the questionnaire from English to these languages. To validate the accuracy of the translations, the questionnaire was then translated back into English by a different team of linguists. Before the commencement of data collection, the lead investigator trained the data collectors and laboratory personnel involved in the study. This training aimed to familiarize them with the study’s objectives, data collection procedures, and laboratory techniques, ensuring consistency and accuracy in data collection and analysis.

To maintain the sterility of the swabs used for sample collection, rigorous checks were conducted, and swab collection followed aseptic techniques, including the use of sterile tubes for transportation, sterile cotton-tipped sticks for swabbing, and clean, powder-free gloves to minimize contamination. Each collected sample was carefully controlled and recorded. Furthermore, quality control measures were implemented during the Gram staining process. Known control slides were used to validate the quality of reagents and the effectiveness of staining procedures. These controls helped ensure the accuracy and reliability of the Gram staining process. Additionally, the performance of the culture medium and antibiotic disks was evaluated using reference strains of *E. coli* (ATCC 259), *S. aureus* (ATCC 25923), and *P. aeruginosa* (ATCC 27853) (30). The turbidity of the bacterial suspension was adjusted to achieve the 0.5 McFarland level for conducting a drug susceptibility test.

### Ethical consideration

The study received ethical clearance from the Institutional Health Research Ethics Review Committee (IHRERC) at Haramaya University, under reference number IHREC/037/2022. Additionally, the Harari Transport Bureau provided an official letter of support for the research. Prior to sample collection, all participants were thoroughly informed about the study’s objectives and voluntarily provided written consent. Strict measures were implemented to ensure the confidentiality of participant information, and positive results were communicated to the respective vehicle drivers. Comprehensive information about the study, including its objectives, methodology, potential risks, and benefits, was shared with each driver involved. Participants were informed of their right to decline participation or withdraw at any point during the research process.

## Results

The study involved 258 vehicles and their drivers, achieving a 100% response rate. Samples were collected from 6 busses, 21 Lada taxis, 22 minibusses, and 209 tricycles. The mean age of the participants was 33.5 years (SD ± 8.3). All participants were male (100%), with a majority in the age group of over 35 years (49.6%). In terms of educational background, 64.3% had completed secondary school, while 12.2% held a college diploma or higher. Over 49.6% of the participants were single. Additionally, 53.1% of the vehicles had more than 300 rides per day, 34.1% had multiple routes, and 54.3% served non-hospital routes (Table 1).

**Table 1:** Sociodemographic characteristics and background variables of intra-city public transport drivers in Harar, Eastern Ethiopia, from 20 June to 30 October, 2023.

Variables	Category	Frequency (*N*)	Percentage (*N* %)
Sex of Participants	Male	258	100.0%
	Female	–	0.0%
Ages of participants	20–24 years	12	4.7%
	25–29 years	85	32.9%
	30–34 years	33	12.8%
	>35 years	128	49.6%
Educational background	Illiterate	-	0.0%
	Primary	30	11.6%
	Secondary	166	64.3%
	Higher education	62	24.0%
Marital status	Single	128	49.6%
	Married	99	38.4%
	Divorced/widower	31	12.0%
Types of taxi (vehicle)	Minibus	22	8.5%
	Lada	21	8.1%
	Tricycle	209	81.0%
	Bus	6	2.3%
Sample location	Doors	57	22.1%
	Handle	87	33.7%
	Seat	114	44.2%
Types of surfaces	Metal	71	27.5%
	Plastic	105	40.7%
	Cloth	82	31.8%
Multiple Route	Not multi-route	170	65.9%
	Multi route	88	34.1%
Hospital route	Not Hospital route	140	54.3%
	Hospital route	118	45.7%
Numbers of passengers per day	1–299	121	46.9%
	>300	137	53.1%
Clean	Regularly cleaned	88	34.1%
	Regularly not cleaned	170	65.9%
Sample collection time	Morning	106	41.1%
	Afternoon	152	58.9%

### Bacteria load from frequently touched surfaces of the vehicles

The total aerobic bacterial load from vehicles (minibusses, Lada taxis, tricycles, and busses) ranged from no growth to 3.05 × 10^6^ CFU/mL, with a mean of 2.994 (SD ± 0.562 × 10^4^ CFU/mL) (Table 2).

**Table 2:** Bacterial load (CFU*10^6^/4cm^2^) from frequently touched surfaces on intra-city public transport in Harar, Eastern Ethiopia, from 20 June to 30 October 2023.

Type of vehicle	Public transport frequently touched surfaces	Pathogenic bacterial contamination level		Pathogenic bacterial isolate		
		Mean	±SD	Yes (*N*)	No (*N*)	Total (*N*)
Minibus (*n* = 22)	Doors	0.709*10^3^	1,503	5	1	6
	Handle	7.173*10^6^	9,356	5	3	8
	Seat	10.625*10^6^	9,171	6	2	8
	Total	6.665*10^6^	8,666	16 (72.7%)	4	22
Lada (*n* = 21)	Doors	0		0	1	1
	Handle	12.376*10^6^	5,641	8	0	8
	Seat	9.709*10^6^	10,483	12	0	12
	Total	10.262*10^6^	8,877	20 (95.2%)	1	21
Tricycle (*n* = 209)	Doors	0.396*10^3^	1,411	23	26	49
	Handle	1.323*10^3^	2,497	52	17	69
	Seat	2.685*10^6^	4,175	76	15	91
	Total	1.699*10^3^	3,303	151 (72.2%)	58	209
Bus (*n* = 6)	Doors	0		0	1	1
	Handle	4.608*10^6^	6,087	2	0	2
	Seat	15.342*10^6^	12,849	3	0	3
	Total	9.207*10^6^	11,020	5 (83.3%)	1	6
Total	Doors	0.415*10^3^	1,387	28 (10.9%)	29	57
	Handle	2.954*10^6^	5,214	67 (25.97%)	20	87
	Seat	4.314*10^6^	5,577	97 (37.6%)	17	114

The majority of vehicle surface samples were found to be contaminated, with an average bacterial load of 2.994 (SD: 0.562 × 10^4^) CFU/4 cm^2^. Significantly higher mean colony counts were observed on the seat surfaces, at 4.314 × 10^6^ (SD: 0.56 ×10^6^) CFU/4 cm^2^ and door surfaces (*p* = 0.0035), at 0.415 × 10^3^ (SD: 0.141 × 10^3^) CFU/cm^2^ compared to the handles, which had 2.954 × 10^6^ (SD: 0.52 × 10^6^) CFU/cm^2^.

### Distributions of pathogenic bacterial isolates by type of vehicles

**Figure 2:**
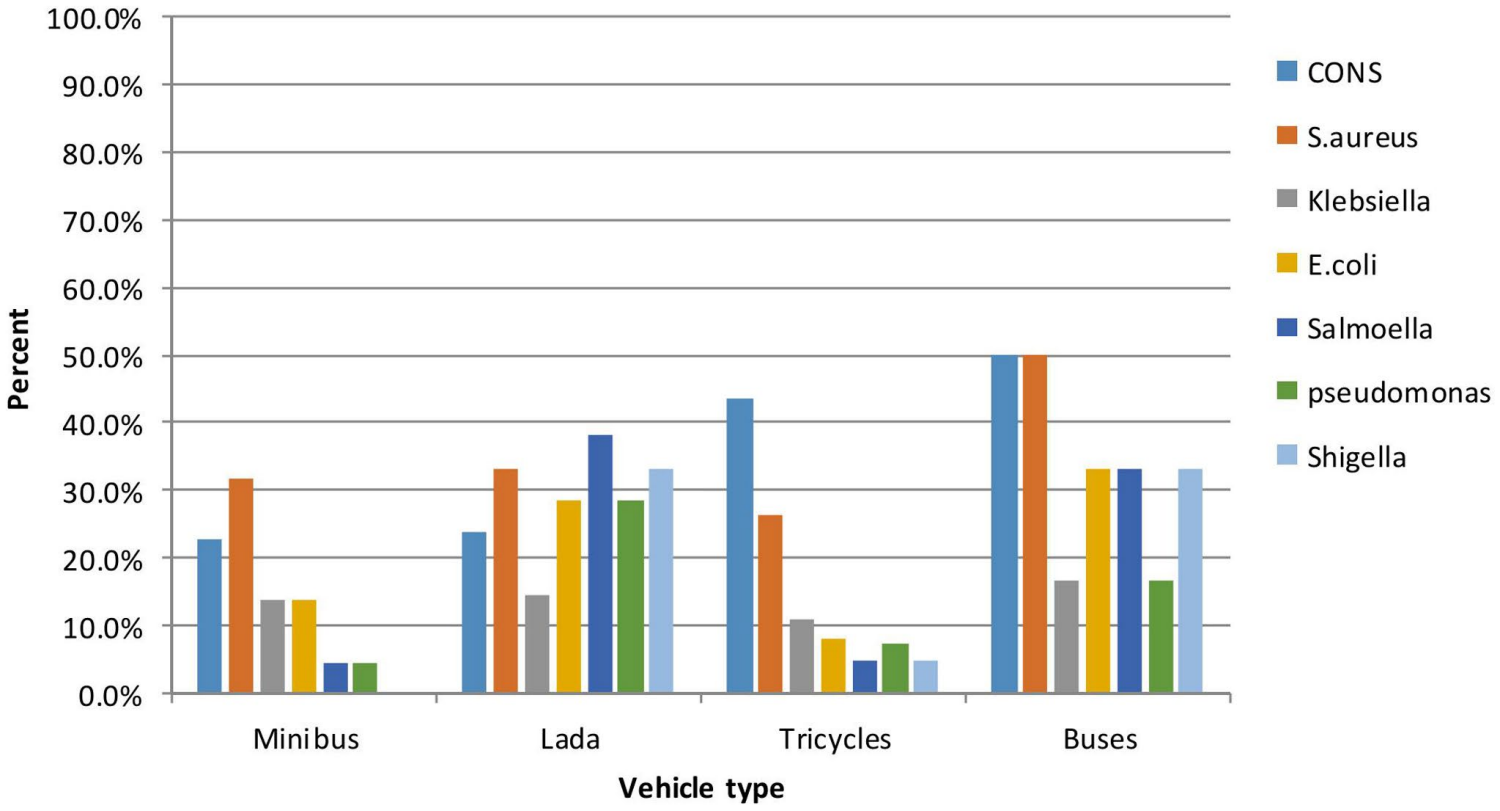
Relative abundance of pathogenic bacteria by type of vehicle of intra-city public transport at Harar, Eastern Ethiopia from June 20 to October 30, 2023.

A total of 258 vehicles were sampled, from which pathogenic bacteria were isolated from 192 vehicles, resulting in an isolation rate of 74.4% (95% CI: 69–80%). Additionally, out of the 364 isolates, 297 were identified as pathogenic bacteria, while the remaining isolates were classified as contaminants. The highest isolation rate was observed in Lada vehicles (95.2%), followed by busses (83.3%), minibusses (72.7%), and tricycles (72.2%) (Table 2). Nearly two-thirds, or 240 out of the 364 bacterial isolates, were Gram-positive, while 124 of them were Gram-negative bacteria. Among the Gram-positive isolates, *coagulase-negative staphylococci* (*CoNS*) were the most frequently isolated (*n* = 104), followed by *Staphylococcus aureus* (*n* = 71), and 65 were Gram-positive bacilli species. Of the 140 Gram-negative isolates, 30 were *Klebsiella species*, 28 were *E. coli*, 23 were *Pseudomonas species*, 21 were *Salmonella species*, and 19 were *Shigella species.* The frequencies of pathogens varied considerably between vehicle types; the bacterial pathogens on bus surfaces included *CoNS* at 3/6 (50.8%), *S. aureus at* 3/6 (50.8%), *E. coli* at 2/6 (33.3%), *Klebsiella species* at 1/6 (16.7%), *Salmonella species* at 2/6 (33.3%), *Shigella species* at 2/6 (33.3%), and *Pseudomonas aeruginosa at* 1/6 (16.7%) (Figure 2).

### Bacterial isolates categorized by vehicle type and specimen collection surface type

The frequencies of bacterial pathogens can also vary considerably between different types of vehicle surfaces. Among the 297 pathogenic bacteria isolated, 151 (50.84%) were found on seats, 110 (37%) on handles, and 36 (12.12%) on doors. Regarding the seats, the breakdown is as follows: *CoNS* 49 (55.7%), *S. aureus* 19 (27.8%), *E. coli* 18 (18.6%), *Klebsiella* spp. 18 (18.6%), *Salmonella* spp. 11 (11.3%), *Shigella* spp. 10 (10.3%), and *Pseudomonas aeruginosa* 13 (13.4%).

### Antimicrobial susceptibility test

A total of 297 pathogenic bacterial isolates were tested against eight different antibiotic disks, revealing higher proportions of resistance rates among Gram-positive bacteria to ampicillin (97.6%) and oxacillin (56.5%). The resistance rate of *S. aureus* to ampicillin (97.6%) and oxacillin (64.1%) is greater than that of CoNS, which shows resistance rates of 95.3% to ampicillin and 40.0% to oxacillin. In contrast, *CoNS* exhibits a higher resistance rate to cefotaxime (44.3%) compared to *S. aureus* (28.2%).

*Escherichia coli* was found to be the most resistant Gram-negative bacterium among the investigated antibiotics: ceftriaxone 64.3% (18/28), ampicillin 94.9% (26/28), chloramphenicol 14.3% (4/28), and cefotaxime 35.7% (10/28). *Klebsiella species* exhibited the highest resistance rates, including ampicillin (100%), ceftriaxone (73.3%), ciprofloxacin (6.6%), cefotaxime (53.3%), and chloramphenicol (26.7%). *Salmonella species* showed resistance to chloramphenicol at 28.7% (6), 61.9% to ceftriaxone, and 38.1% to cefotaxime. *Shigella species* exhibited resistance to ceftriaxone (61.9%), cefotaxime (38.1%), and chloramphenicol (28.7%). The resistance rate in *Pseudomonas* spp. was 100% to ampicillin, 54.5% to ceftriaxone, 41% to cefotaxime, and 38.1% to chloramphenicol (Table 3).

**Table 3:** Patterns of antibiotic resistance in bacterial isolates from frequently touched surfaces of intra-city public transport in Harar, Eastern Ethiopia, from June to October 2023.

Antibiotic resistance patterns	*CONS*			*S. aureus*			*E. coli*			*Klebsiella* spp.			*Pseudomonas* spp.			*Salmonella* spp.			*Shigella* spp.		
	S	I	R	S	I	R	S	I	R	S	I	R	S	I	R	S	I	R	S	I	R
Ampicillin	0	23	91	0	2	68	0	2	26	0	0	29	0	2	19	0	1	21	0	1	20
Oxacillin	47	23	34	21	9	41	–	–	–	–	–	–	–	–	–	–	–	–	–	–	–
Erythromycin	76	19	9	58	4	9	–	–	–	–	–	–	–	–	–	–	–	–	–	–	–
Chloramphenicol	103	1	0	64	2	5	22	2	4	21	0	8	15	2	4	21	0	2	20	0	2
Gentamicin	104	0	0	71	0	0	28	0	0	29	1	0	21	0	0	23	0	0	22	0	0
Ceftriaxone	–	–	–	–	–	–	3	7	18	8	7	15	8	3	10	9	0	12	6	4	12
Ciprofloxacin	102	2	0	71	0	0	27	1	0	26	2	2	21	0	0	23	0	0	22	0	0
Cefotaxime	68	0	46	36	15	20	12	6	10	14	4	12	13	3	5	7	5	10	11	1	10

I, intermediate; R, resistance; and S, susceptibility.

**Figure 3:**
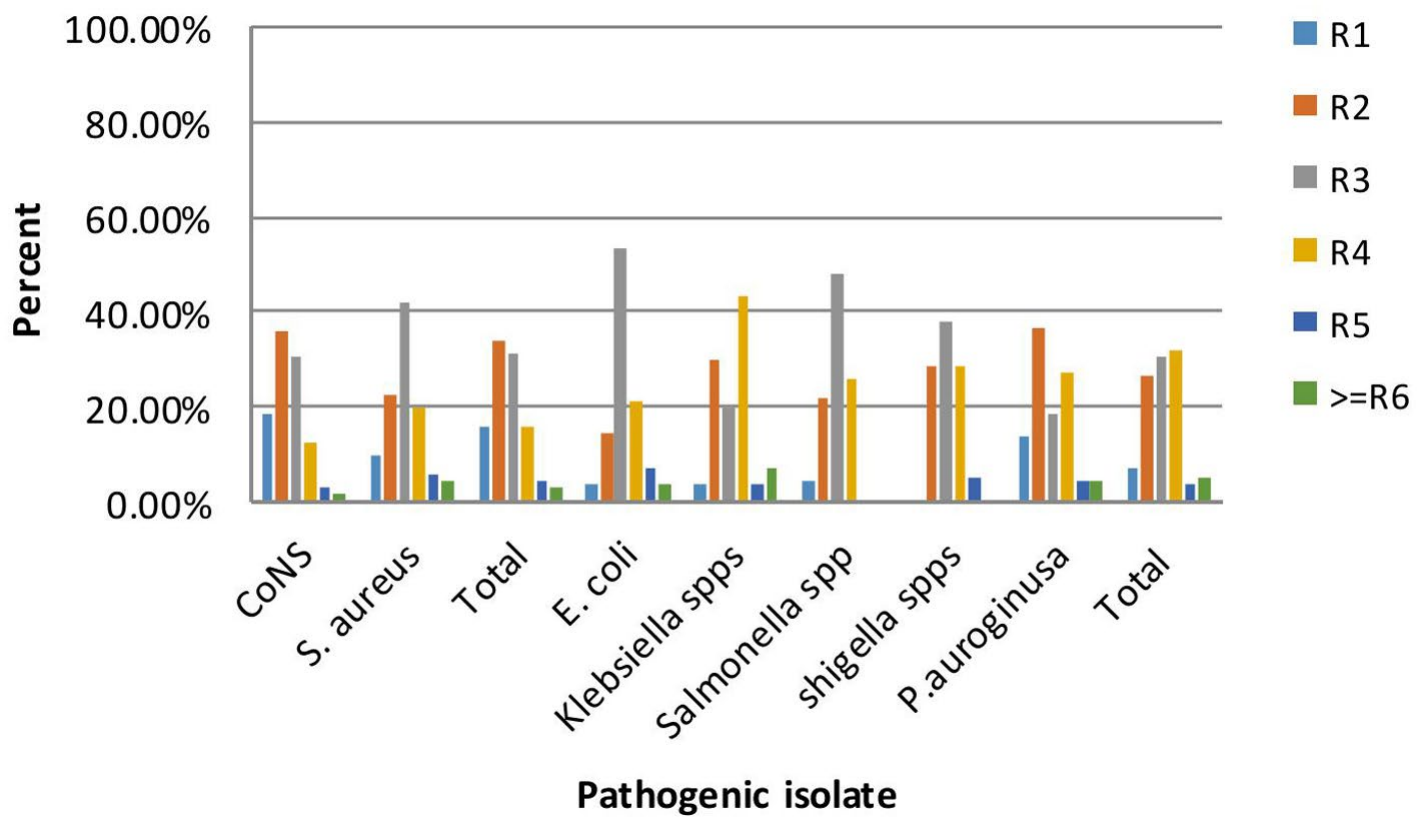
Multi-drug resistance patterns of bacterial isolate on frequently touched surfaces of intra-city public transport at Harar, Eastern Ethiopia from June 20 to October 30, 2023.

The antibiogram of Gram-positive bacterial isolates (15.6%) did not show resistance to any of the seven antibiotic classes tested (ampicillin, oxacillin, erythromycin, chloramphenicol, gentamicin, ceftriaxone, ciprofloxacin, and cefotaxime); however, none of the isolates exhibited resistance to all the antibiotics tested. MDR was observed in 67 out of 297 (22.5%) isolates. Notably, a higher rate of MDR was detected in 34 out of 121 (28.1%) Gram-negative isolates. More frequently, 53.6% of *E. coli* were multidrug-resistant, followed by *Klebsiella species* at 43.3%. Overall, among the total isolates (*n* = 67/297), multidrug resistance, defined as resistance to ≥3 antibiotic classes, is shown in Figure 3.

### Multiple linear regression analysis

A multiple linear regression analysis was conducted to determine whether the independent variables are related to bacterial load. Handles: (*β* = 1.18, *t* = 6.602, *p* < 0.001), seats: (*β* = 1.65, *t* = 9.56, *p* < 0.001), cloth surfaces: (*β* = 0.433, *t* = 2.451, *p* < 0.015), multi-route services: (*β* = 0.496, *t* = 3.36, *p* < 0.001), more than 300 passengers per day: (*β* = 0.57, *t* = 4.036, *p* < 0.001), and regularly not cleaned: (*β* = 0.497, *t* = 3.521, *p* < 0.001) are positively associated with bacterial load. This suggests that tricycles, handles, the number of passengers per day ≥300, regularly not cleaned, multi-route services, cloth surfaces, and seats play a pivotal role in predicting CFU. Meanwhile, the results also indicate that busses: (*β* = 0.876, *t* = 1.83, *p* < 0.069), Lada (*β* = 0.367, *t* = 1.13, *p* < 0.259), plastic surfaces (*β* = 0.083, *t* = 0.51, *p* < 0.610), and hospital routes (*β* = 0.055, *t* = 0.400, *p* < 0.690) do not impact bacterial loads (Table 4).

**Table 4:** Multi-linear regression of log^10^ bacterial load on frequently touched surfaces of intra-city public transport in Harar, Eastern Ethiopia, from 20 June to 30 October 2023.

Variable		Unstandardized Coefficients		*T*	95.0% Confidence Interval for B		Sign	Beta
		B	Std. error		Lower bound	Upper bound		
		0.851	0.275	3.092	0.309	1.393	0.002	
Types of taxi	Minibus						1	
	Lada	0.367	0.324	1.131	−0.272	1.006	0.259	0.069
	Tricycle	−0.720	0.235	−3.069	−1.183	−0.258	0.002	−0.195
	Bus	0.876	0.480	1.825	−0.069	1.821	0.069	0.091
Sample location	Doors						1	
	Handle	1.181	0.179	6.602	0.829	1.534	0.000	0.386
	Seat	1.651	0.173	9.554	1.311	1.992	0.000	0.567
Types of surfaces	Metal						1	
	Plastic	0.083	0.163	0.511	−0.238	0.405	0.610	0.028
	Cloth	0.433	0.177	2.451	0.085	0.781	0.015	0.139
Multiple route	No						1	
	Yes	0.496	0.148	3.356	0.205	0.787	0.001	0.163
Hospital route	No						1	
	Yes	0.055	0.138	0.400	−0.216	0.326	0.690	0.019
Passengers	1–299						1	
	>300	0.565	0.140	4.036	0.289	0.841	0.000	0.195
Regularly Cleaning	Yes						1	
	No	0.497	0.141	3.521	0.219	0.775	0.001	0.163
Sample collection time	Morning						1	
	Afternoon	0.301	0.138	0.029	0.574	0.103	0.030	2.181

There was a statistically significant difference in the multiple routes (*p* < 0.001), the number of passengers greater than 300 per day (*p* < 0.001), and those that are regularly uncleaned (*p* < 0.001). The tolerance (0.911) and variance inflation factor (2.057) values did not indicate a violation of this assumption. The Durbin–Watson statistic was calculated to assess the assumption that the value of the residuals is independent, suggesting that this assumption was not violated (1.17).

### Bivariate and multivariate logistic regression

In the bivariate analysis, factors such as marital status, type of taxi (vehicle), sample location, presence of multiple routes, hospital routes, daily passenger count, regular cleaning practices, and sample collection time were considered for multivariable logistic regression with a *p-*value of <0.25. The subsequent multivariable logistic regression analysis identified several significant variables associated with pathogenic bacteria contamination of vehicle surfaces, including sample locations on handles and seats, the presence of multiple routes, hospital routes, a daily passenger count exceeding 300, regular uncleanliness, and sample collection time in the afternoon, all with *p*-values <0.05.

The multivariable analysis revealed that sample locations from vehicle handles were 2.5 times more likely to be contaminated with pathogenic bacteria compared to door surfaces (AOR: 2.5; 95% CI: 1.1, 6.2). Seats exhibited a five-fold increase in contamination by pathogenic bacteria compared to door surfaces (AOR: 5; 95% CI: 2.1, 12). Vehicles operating on multiple routes had contamination levels from pathogenic bacteria that were 3.7 times higher than those that did not (AOR: 3.7; 95% CI: 1.4, 9.7), while vehicles on hospital routes were 2.36 times more likely to be contaminated by pathogenic bacteria compared to those that were not (AOR: 2.36; 95% CI: 1.1, 5.1). Furthermore, vehicles accommodating more than 300 passengers per day exhibited 2.2 times greater contamination than those with fewer passengers (AOR: 2.2; 95% CI: 1.2, 4.5). Regularly uncleaned vehicle surfaces were 3.6 times more contaminated than clean surfaces (AOR: 3.6; 95% CI: 1.7, 7.3). In addition, vehicles sampled in the afternoon were 3.5 times more likely to be contaminated than those sampled in the morning (AOR: 3.5; 95% CI: 1.7, 7). These findings highlight the significant associations between vehicle characteristics, usage patterns, and the risk of pathogenic bacterial contamination (Table 5).

**Table 5:** Bivariate and multivariable logistic regression of pathogenic bacteria on frequently touched surfaces of intra-city public transport in Harar, Eastern Ethiopia, from 20 June to 30 October 2023.

Characteristics	Pathogenic bacteria		Crude OR (95% CI)	Adjusted OR (95% CI)	*p*-value
	Yes (%)	No (%)			
Marital status					
Single	89 (69.5%)	39 (30.5%)			1
Married	77 (77.8%)	22 (22.2%)	1.53 (0.84, 2.81)	1.78 (0.79, 3.96)	0.159
Divorced/w	26 (83.9%)	5 (16.1%)	2.28 (0.81, 6.37)	2.5 (0.73, 8.69)	0.145
Educational background					
Primary	21 (70%)	9 (30%)			1
Secondary	124 (74.7%)	42 (25.3%)	1.26 (0.54, 2.98)		0.555
Higher	47 (75.8%)	15 (24.2%)	1.34 (0.50, 3.55)		0.553
Types of surfaces					
Metal	52 (74.6%)	18 (25.4%)			1
Plastic	77 (73.3%)	28 (26.7%)	0.93 (0.47, 1.86)		0.846
Cloth	62 (75.6%)	20 (24.4%)	1.05 (0.50, 2.19)		0.891
Types of taxi					
Minibus	16 (72.7%)	6 (27.3%)			1
Lada	20 (95.2%)	1 (4.8%)	7.5 (0.82, 68.8)	1.4 (0.11, 17.1)	0.789
Tricycle/Bajaj	151 (72.2%)	58 (27.8%)	0.97 (0.36, 2.62)	0.57 (0.17, 1.89)	0.361
Bus	5 (83.3%)	1 (16.7%)	1.87 (0.18, 19.52)	1.47 (0.08, 25.6)	0.791
Sample location					
Door	28 (49.1%)	29 (50.9%)			
Handle	67 (77%)	20 (23%)	3.47 (1.69, 7.13)	2.57 (1.06, 6.2)	0.036*
Seat	97 (85.1%)	17 (14.9%)	5.91 (2.84, 12.28)	5.0 (2.08, 12)	0.0000*
Multiple route					0.004
No	111 (65.3%)	59 (34.7%)			1
Yes	81 (92%)	7 (8%)	6.15 (2.67, 14.16)	3.67 (1.39, 9.72)	0.009*
Hospital route					
No	91 (65%)	49 (35%)			1
Yes	101 (85.6%)	17 (14.4%)	2.39 (1.19, 4.81)	2.36 (1.09, 5.08)	0.028*
Numbers of passengers per day					
1–299	74 (61.2%)	47 (38.8%)			1
>300	118 (86.1%)	19 (13.9%)	3.94 (2.15, 7.24)	2.19 (1.06, 4.54)	0.035*
Regularly cleaning					0.00023
Yes	49 (75.7%)	39 (44.3%)			1
No	143 (84.1%)	27 (15.9%)	4.21 (2.34, 7.59)	3.56 (1.74, 7.28)	0.001*
Sample collection time					
Morning	60 (56.6%)	46 (43.4%)			1
Afternoon	132 (86.8%)	20 (13.2%)	5.06 (2.76, 9.29)	3.45 (1.69, 7.02)	0.001*

^*^Statistically significant at a *p*-value of <0.05 level.

## Discussion

This study examines the prevalence of pathogenic bacteria contamination in public transportation and its implications for public health. The current results reveal that 74.4% (95% CI: 69–80%) of public transport vehicles harbor pathogenic bacteria. However, this rate is higher than findings from Tigray, Ethiopia (22%) (1), Addis Ababa, Ethiopia (28.9%) (5), and New York City (46.9%) (31). This discrepancy may be due to various factors, such as geographical location, the number of passengers per day, frequency and types of routes, cleaning protocols, and public sanitation awareness (16, 32). Consistent with findings from India (80%) (33), this rate is lower than the results reported from Nigeria (84%) (34), Lebanon (91%) (35), and Bangladesh (100%) (9), possibly due to differences in cleaning protocols and public sanitation awareness.

The predominant bacterial isolates in this study were *coagulase-negative staphylococci* (*CoNS*; 40.3%), likely reflecting its preponderance on normal skin (36). Consecutively, *S. aureus* (27.5%), Gram-positive *Bacilli* (25.2%), *Klebsiella* spp. (11.6%), *E. coli* (10.9%), *Pseudomonas* spp. (8.9%), *Salmonella* spp. (8.3%), and *Shigella* spp. (7.4%) were isolated.

These findings align with earlier research in Ethiopia (1) and other countries, such as Nepal and India (33, 37). However, pathogens such as *Listeria* and *Serratia*, which were reported in studies from Nigeria (34), were not identified in this study. In addition, some bacterial species detected in other studies were not isolated in this study.

*CoNS*, with a prevalence of 40.3%, was frequently isolated from transport surfaces, similar to findings in Indonesia (44.54%) (38) and the USA (43%) (39). *S. aureus*, identified in 27.5% of samples, showed isolation rates consistent with studies from Kenya (33%) (26) and Indonesia (27.4%) (38). This bacterium is known for colonizing mucous membranes in humans (31). However, this rate was lower than those reported from Lebanon (58.6%) (35) and the USA (68%) (30). These differences could be due to variations in microbiological analysis methods (40).

The third most common isolate, Gram-positive *Bacilli* (25.2%), was consistent with findings from Nigeria (20.5%) (41) and Poland (20–29%) (10). *Klebsiella* spp. (11.6%) and *E. coli* (10.9%) were also significant contaminants linked to poor hygiene practices among passengers and drivers, as observed in studies from Kenya (24%) (26)and India (14%) (33). *Pseudomonas* spp. (8.9%) and *Salmonella* spp. (8.3%) were other notable isolates, consistent with global studies but varying in prevalence due to environmental factors and sanitation standards.

Antimicrobial resistance profiles revealed high resistance to ampicillin (97.6%) and oxacillin (56.5%) among Gram-positive bacteria, including *CoNS* and *S. aureus*. *CoNS* showed higher resistance to cefotaxime (44.3%) compared to *S. aureus* (28.2%). These results align with other studies (42, 43), indicating that bacterial resistance is an evolving phenomenon arising from genetic mutations and/or acquired genomes (44). Gentamicin remained the most effective treatment, showing 88.6% efficacy and aligning with studies conducted in Kenya (26). However, multidrug resistance was observed in 22.5% of isolates, emphasizing the need for stricter antibiotic usage protocols and hygiene practices in public transport environments.

Gram-negative bacteria also exhibited high resistance rates, with over 95% showing resistance to at least one class of antibiotics, including *E. coli* and *Klebsiella* spp. This demonstrates significant resistance and underscores the challenges of treating infections linked to contaminated transport surfaces. The high prevalence of multidrug-resistant organisms indicates that the irrational use of antibiotics and inadequate infection control measures are contributing factors.

Contamination levels varied significantly across different transport modes and surfaces, with tricycles demonstrating 72% lower pathogen bacterial loads (*β* = −0.720, *t* = −3.069, *p* < 0.001), possibly due to improved ventilation. In contrast, busses and minibusses provide favorable environments for microbial growth. These findings are consistent with studies from Ghana, where busses exhibited significantly higher bacterial loads compared to other transport types (25).

Contamination was significantly higher on seats and handles compared to doors, with bacterial loads reaching 4.31 ± 0.56 × 10^6^ CFU/4 cm^2^ on seats (*p* < 0.001). Materials such as cloth and polythene, commonly used in vehicle interiors, were associated with higher bacterial counts than metal surfaces, which are easier to clean. This observation aligns with studies from Germany, highlighting the impact of material type on bacterial load (45). Multiple routes for service vehicles showed 3.7 times increased odds of bacterial contamination (AOR: 3.7; *p* < 0.001), and vehicles with more than 300 riders per day had 2.2 times increased odds of bacterial contamination and bacterial loads (AOR: 2.2; *p* < 0.05; *β* = 0.57, *p* < 0.001). Similar to studies conducted in Iran, the number of passengers (*r* = 0.518, *p* < 0.001) (46). A study published in a report from a Midwestern US city found a strong relationship between disease-causing bacteria and the population (25). However, contrary to the American ridership study (*p* < 0.088), the results for multiple routes were not significant (*p* < 0.058) (29). A possible reason for this is overcrowding. Regularly uncleaned vehicles had 3.6 times increased odds of bacterial contamination (AOR: 3.6; *p* < 0.001). This finding is consistent with studies conducted in Kenya (26). A possible explanation could be the lack of a regular sanitation program.

The study has several possible weaknesses. First, it was limited to aerobic bacteria, thus excluding anaerobic and non-cultivable pathogenic bacteria. Additionally, observational bias may have occurred during cleaning practices, and antibiotic susceptibility tests were not conducted on Gram-positive rod isolates. Second, although generalizability was a goal, the results of this study might not be fully applicable in other contexts. There is no information regarding the rates of bacterial colonization or local illnesses in the research area. Therefore, anticipated national averages should be taken into consideration when interpreting the results of this study. Finally, due to resource limitations and the overall goals of the study, only a small number of isolates were examined at each stage of the investigation, although not at the gene level.

## Conclusion

The study reveals significant contamination from pathogenic bacteria in public transport, indicating the potential role of these intra-city vehicles in disease transmission. Our findings show a high diversity of bacterial species across all vehicles. Despite identifying microorganisms on the surfaces of these vehicles, predicting whether people will ultimately be affected by pathogenic bacterial infections is challenging. Seats were found to be the most contaminated surfaces, followed by handles. Gram-positive bacteria, particularly *CoNS* and *S. aureus,* comprised half of the isolates, while Gram-negative bacteria such as *Klebsiella* sp.*, E. coli, Pseudomonas* sp., and *Salmonella* sp. were also prevalent. The study showed a high rate of drug resistance to common antimicrobial agents. Factors contributing to contamination included vehicle type, surface material, multi-route service, high ridership, and poor cleaning. Regional health authorities, health professionals, transport authorities, and other stakeholders should collaborate to improve awareness among drivers, vehicle owners, and the community about the potential health risks of infectious diseases related to public transport. Environmental health should collaborate with transport authorities to ensure regular cleaning inspections and improved control measures.

## Data Availability

The original contributions presented in the study are included in the article/supplementary material. Further inquiries can be directed to the corresponding author.

## References

[1] Kahsay AG Asgedom SW Weldetinsaa HL. Enteric bacteria, methicillin resistant *S. aureus* and antimicrobial susceptibility patterns from buses surfaces in Mekelle city, Tigray, Ethiopia. BMC Res Notes. (2019) 12:1–5. doi: 10.1186/s13104-019-4366-1 31196155 PMC6567901

[2] Iles R. Public transport vehicle types, public transport in developing countries Emerald Group Publishing Limited Book, English, 1st edn. Amsterdam: Elsevier (2005).

[3] Champahom T Jomnonkwao S Karoonsoontawong A Hantanong N Beeharry R Ratanavaraha V. Modeling user perception of bus service quality: case study of Mauritius. Songklanakarin J Sci Tech. (2019). doi: 10.3390/su12104259

[4] Angbuhang KB Neupane M Adhikari A Binita K Jha S. Detection of methicillin resistant *Staphylococcus aureus* in public transportation of Kathmandu Valley, Nepal. Tribhuvan university. J Microbiol. (2018) 5:51–6. doi: 10.3126/tujm.v5i0.22312, PMID: 37609976

[5] Akele M. Bacterial profiles and antibacterial susceptibility patterns of frequently hand touched surfaces on public transport buses, automated teller machines and St’ Paul Hospital patient waiting areas in Addis Ababa, Ethiopia June, 2019. Addis Ababa, Ethiopia: Ethiopian Journal of Public Health and Nutrition (EJPHN). (2019).

[6] Huang L Xu C Yang W Yu R. A machine learning framework to determine geolocations from metagenomic profiling. Biol Direct. (2020) 15:1–12. doi: 10.1186/s13062-020-00278-z 33225966 PMC7682025

[7] Atienza DM De Leon AJF Pineda CJM Santiago JAA Sardoma GRB Anastacio JM. Investigation of interior air quality of taxicabs using LPG In:. Mapua Institute of Technology undergraduate thesis. Proceedings of the 18th Annual Conference of the Transportation Science Society of the Philippines (2010).

[8] Orogu J. Ehiwario N. Okobia U., Microbiological assessment of the pedestrian hand rails of delta state polytechnic, Ozoro. J. Pharm. Innov. (2018) 7:581–58.

[9] Chowdhury T Mahmud A Barua A Khalil MI Chowdhury R Ahamed F. Bacterial contamination on hand touch surfaces of public buses in Chittagong City, Bangladesh. IOSR J Environ Sci Toxicol Food Technol. (2016) 10:48–51. doi: 10.9790/2402-1009024851

[10] Medveďová A Györiová R. Prevalence of Staphylococcus aureus and antibiotic resistant *Staphylococcus aureus* in public transport in Bratislava, Slovakia. Acta Chimica Slovaca. (2019) 12:41–5. doi: 10.2478/acs-2019-0007

[11] Sharafi K Khodadadi T Khosravi T Moradi M. Investigation of microbial contamination frequency in drinking water of buses at Sofeh terminal of Isfahan-Iran. Int. J. Environ. Health Eng. (2015) 4:15. doi: 10.4103/2277-9183.157711

[12] Friedman L Wallar LE Papadopoulos A. Environmental risk factors for community-acquired MRSA National Collaborating Center for Environmental Health. Public university in Guelph, Canada: University of Guelph (2015).

[13] Domon H Uehara Y Oda M Seo H Kubota N Terao Y. Poor survival of methicillin-resistant *Staphylococcus aureus* on inanimate objects in the public spaces. Microbiology. (2016) 5:39–46. doi: 10.1002/mbo3.308, PMID: 26503447 PMC4767431

[14] Nworie A Ayeni J Eze U Azi S. Bacterial contamination of door handles/knobs in selected public conveniences in Abuja metropolis, Nigeria: a public health threat. Cont J Med Res. (2012) 6:7–11.

[15] Zou Z-Y Lei L Chen Q-Y Wang Y-Q Cai C Li W-Q. Prevalence and dissemination risk of antimicrobial-resistant Enterobacteriaceae from shared bikes in Beijing, China. Environ Int. (2019) 132:105119. doi: 10.1016/j.envint.2019.105119, PMID: 31491607

[16] Ly Y-T Leuko S Moeller R. An overview of the bacterial microbiome of public transportation systems—risks, detection, and countermeasures. Front Public Health. (2024) 12:1367324. doi: 10.3389/fpubh.2024.1367324, PMID: 38528857 PMC10961368

[17] Tatem AJ Rogers DJ Hay SI. Global transport networks and infectious disease spread. Adv Parasitol. (2006) 62:293–343. doi: 10.1016/S0065-308X(05)62009-X, PMID: 16647974 PMC3145127

[18] Ncama BP Kuupiel D Duma SE Mchunu G Guga P Slotow R. Scoping review of food safety at transport stations in Africa. BMJ Open. (2021) 11:e053856. doi: 10.1136/bmjopen-2021-053856, PMID: 34824120 PMC8627411

[19] John OU Adegoke AA. Bacteriological evaluation of hand contact surfaces at bus terminals in Uyo Metropolis. J Pure Appl Microbiol. (2018) 12:1187–93. doi: 10.22207/JPAM.12.3.18

[20] Jifar A Ayele Y. Assessment of knowledge, attitude, and practice toward antibiotic use among Harar city and its surrounding community, eastern Ethiopia. Interdiscip Perspect Infect Dis. (2018) 2018:1–6. doi: 10.1155/2018/8492740, PMID: 30174690 PMC6106796

[21] Tamiru C Abdela T. Assessment of on-farm diversity status and Farmer’s perception for landraces crops at eastern Hararghe, Ethiopia. Agric Forest Fish. (2021) 10:152. doi: 10.11648/j.aff.20211004.15

[22] Fufa DD Abhram A Awgichew Teshome KT Abera F MaledaTefera MY Mengistu M. Hygienic practice of complementary food preparation and associated factors among mothers with children aged from 6 to 24 months in rural kebeles of Harari region, Ethiopia. Food Sci Technol. (2020) 8:34–42. doi: 10.13189/fst.2020.080203

[23] Beareu H.T. Service of vehicle or taxi, Harar town in transport Beareu: Harar, Regional State (2013). Available at: https://arifchereta.com/tenders/harari-transport-and-roads-development-bureau-now-invites-bidders-necessary-labor-materials-and-equipment-for-the-construction-works-of-different-projects/ (Accessed September 16, 2024).

[24] Hurst CJ Crawford RL Garland JL Lipson DA. Manual of environmental microbiology American Society for Microbiology Press. USA: University and Universidad del Valle (2007).

[25] Abdulai M Abubabakari Z Cobinna S Oduro D. BACTERIA loads of public transport in the tamale metropolis, Ghana. UDS Int J Develop. (2020) 7:379–86. doi: 10.47740/492.UDSIJD6i

[26] Chebon S Sonoiya JC. Diversity and antibiotics susceptibility of bacterial species on hand surfaces in public buses plying Kenyatta National Hospital Route 7c in Nairobi. Microbiol Res J Int. (2019) 27:1–9. doi: 10.9734/mrji/2019/v27i230096

[27] Ikala R Olayinka B Ehinmidu J. Characterization of *Staphylococcus aureus* small colony variant (scv) clinical isolates in Zaria, Nigeria. Ann Res Rev Biol. (2018) 30:1–9. doi: 10.9734/arrb/2018/v30i630029, PMID: 30315521

[28] Saadat S Solhjoo K Norooz-Nejad M-J Kazemi A. VanA and vanB positive vancomycin-resistant *Staphylococcus aureus* among clinical isolates in shiraz, south of Iran. Oman Med J. (2014) 29:335–9. doi: 10.5001/omj.2014.90, PMID: 25337309 PMC4202220

[29] Lutz JK Van Balen J Mac Crawford J Wilkins JR III Lee J Nava-Hoet RC. Methicillin-resistant *Staphylococcus aureus* in public transportation vehicles (buses): another piece to the epidemiologic puzzle. Am J Infect Control. (2014) 42:1285–90. doi: 10.1016/j.ajic.2014.08.016, PMID: 25465258

[30] CLSI. Performance standards for antimicrobial susceptibility testing. M100. 30th ed (2020). 40 p. Available at: https://www.nih.org.pk/wp-content/uploads/2021/02/CLSI-2020.pdf (Accessed January 20, 2024).

[31] Afshinnekoo E Meydan C Chowdhury S Jaroudi D Boyer C Bernstein N. Geospatial resolution of human and bacterial diversity with city-scale metagenomics. Cell Syst. (2015) 1:72–87. doi: 10.1016/j.cels.2015.01.001, PMID: 26594662 PMC4651444

[32] Haas CN Rose JB Gerba CP. Quantitative microbial risk assessment. Hoboken, New Jersey: John Wiley & Sons (2014).

[33] Shishodia A Joshi A Sharma M. Prevalence and biochemical characterization of Bacteria isolated from some river/canal Bank water sources, door of transport vehicles and shop counters of Western UP and Uttrakhand area in India. J Pure Appl Sci Technol. (2015) 5:1–81.

[34] Viancelli A Fornari BF Fonseca TG Mass AP Ramos FM Michelon W. Contamination, by pathogenic multidrug resistant bacteria on interior surfaces of ambulances. Res Soc Dev. (2022) 11:e48111225925–5. doi: 10.33448/rsd-v11i2.25925

[35] Iskandar S Saif A Nawas T. Isolation of potentially pathogenic bacteria from public service cars door handles. Int J Curr Microbiol App Sci. (2018) 1:1154–9. doi: 10.20546/ijcmas.2018.712.142

[36] Becker K Heilmann C Peters G. Coagulase-negative staphylococci. Clin Microbiol Rev. (2014) 27:870–926. doi: 10.1128/CMR.00109-13, PMID: 25278577 PMC4187637

[37] Sapkota S Khadka S Adhikari S Parajuli A Kandel H Regmi RS. Microbial diversity and antibiotic susceptibility pattern of bacteria associated with motorcycle helmets. Int J Microbiol. (2020) 2020:1–7. doi: 10.1155/2020/8877200, PMID: 33488730 PMC7803264

[38] El Jannah SM Rahayu C Zuraida Z Prasetio R Sugiarto RI. Preliminary research: identification of microorganism in the waiting room on public transportation facilities, DKI Jakarta. SANITAS Jurnal Teknologi Dan Seni Kesehatan. (2017) 8:9–15. doi: 10.36525/sanitas.2017.2

[39] Stephenson RE Gutierrez D Peters C Nichols M Boles BR. Elucidation of bacteria found in car interiors and strategies to reduce the presence of potential pathogens. Biofouling. (2014) 30:337–46. doi: 10.1080/08927014.2013.873418, PMID: 24564823 PMC3962071

[40] Frankel M Timm M Hansen E Madsen AM. Comparison of sampling methods for the assessment of indoor microbial exposure. Indoor Air. (2012) 22:405–14. doi: 10.1111/j.1600-0668.2012.00770.x, PMID: 22299641

[41] AL-Harmoosh AR Eidan AJ Al-Hadrawy HA Mohammed QA Hamed AQ. Potential bacterial contaminants in the handles of Car doors. J Pure Appl Microbiol. (2018) 12:2193–8.

[42] Gahimbare L Muvunyi CM Guessennd NAK Rutanga JP Gashema P Fuller W. Antimicrobial resistance in the WHO African region: a systematic literature review 2016–2020. Antibiotics. (2024) 13:659. doi: 10.3390/antibiotics13070659, PMID: 39061341 PMC11273377

[43] Gebremariam NM Bitew A Tsige E Woldesenbet D Tola MA. A high level of antimicrobial resistance in gram-positive cocci isolates from different clinical samples among patients referred to Arsho advanced medical laboratory, Addis Ababa, Ethiopia. Infect Drug Resist. (2022) 15:4203–12. doi: 10.2147/IDR.S372930, PMID: 35946034 PMC9357381

[44] Jubeh B Breijyeh Z Karaman R. Resistance of gram-positive bacteria to current antibacterial agents and overcoming approaches. Molecules. (2020) 25:2888. doi: 10.3390/molecules25122888, PMID: 32586045 PMC7356343

[45] Gołofit-Szymczak M Stobnicka-Kupiec A Górny RL. Impact of air-conditioning system disinfection on microbial contamination of passenger cars. Air Qual Atmos Health. (2019) 12:1127–35. doi: 10.1007/s11869-019-00731-7

[46] Naddafi K. Jabbari H. Hoseini M. Nabizade R. Rahbar M. Yunesian M. (2011). Investigation of indoor and outdoor air bacterial density in Tehran subway system. In Iranian Journal of Environmental Health Science & Engineering.

